# Three Nutritional Indices Are Effective Predictors of Mortality in Patients With Type 2 Diabetes and Foot Ulcers

**DOI:** 10.3389/fnut.2022.851274

**Published:** 2022-03-15

**Authors:** Jing Hong, Qi-Qi Huang, Wen-Yue Liu, Xiang Hu, Fei-Fei Jiang, Ze-Ru Xu, Fei-Xia Shen, Hong Zhu

**Affiliations:** ^1^Department of Endocrinology, The First Affiliated Hospital of Wenzhou Medical University, Wenzhou, China; ^2^Department of Nutrition, The First Affiliated Hospital of Wenzhou Medical University, Wenzhou, China

**Keywords:** diabetes mellitus, foot ulcer, malnutrition, mortality, nutrition assessment

## Abstract

**Introduction:**

Malnutrition has been associated with mortality in various diseases. This retrospective cohort study aimed to investigate the relationship between three nutritional indices and all-cause mortality in patients with diabetic foot ulcers (DFUs).

**Materials and Methods:**

A total of 771 patients diagnosed with DFUs in the First Affiliated Hospital of Wenzhou Medical University from 2015 to 2019 were included in this retrospective cohort study. Patients were classified as high nutritional risk groups or low nutritional risk groups according to the optimal cut-off values of the geriatric nutritional risk index (GNRI), prognostic nutritional index (PNI), and controlling nutritional status (CONUT), respectively. The associations of three nutritional indices with all-cause mortality were evaluated by multivariable Cox regression analyses.

**Results:**

Log-rank tests indicated that patients with high nutritional risk had lower overall survival rates (all *p* < 0.001). The multivariable Cox regression revealed that low GNRI (adjusted HR 2.01, 95% CI: 1.37–2.96, *P* < 0.001), low PNI (adjusted HR 2.04, 95% CI: 1.29–3.23, *P* = 0.002) and high CONUT (adjusted HRs 1.54, 95% CI: 1.07–2.23, *P* = 0.021) were independently associated with high all-cause mortality. In subgroup analyses, only GNRI predicted higher all-cause mortality in patients with severe DFUs, while all of the three indices persisted as independent prognostic factors in patients with no severe DFUs.

**Discussion:**

The present study demonstrated that three nutritional indices were effective predictors of all-cause mortality in patients with DFUs. Routine screening for malnutrition using any of the three nutritional indices might be a simple and effective way to identify high-risk patients with DFUs. GNRI can be used as an independent prognostic indicator in patients with severe DFUs.

## Introduction

Patients with diabetic foot ulcers (DFUs) are considered to be with an excess risk of all-cause mortality (1), facing a 5-year mortality as high as 30.5% which is comparable to cancer (2). Additionally, the mortality of patients with DFUs is more than 2-folds higher than patients with diabetes but without DFUs (3). The excess all-cause mortality in patients with DFUs cannot fully be explained by traditional cardiovascular risk factors (4). The importance of other factors, such as nutritional status, needs to be further elucidated.

Patients with DFUs, especially those with Wagner grade 4 and 5, were more vulnerable to malnutrition compared to patients without DFUs (5). Malnutrition was found to be associated with higher complications, longer hospital stays, and increased mortality in hospitalized patients (6). Malnutrition is often ignored but modifiable. Identifying patients at risk of malnutrition is important. They might benefit from clinical nutritional interventions. Then improve their outcomes and prolong life (7). There are many screening tools for malnutrition, among them, the geriatric nutritional risk index (GNRI) (8), the prognostic nutritional index (PNI) (9), and the controlling nutritional status (CONUT) index (10) are relatively simple, convenient, effective and practical. They can be calculated from inexpensive and easily-obtained parameters: albumin (ALB), total cholesterol (TC), lymphocyte count, height, and weight.

Literature concerning the association of nutritional index with the prognosis of DFUs is sparse (11). Therefore, in this study, we aimed to explore the role of GNRI, PNI, and CONUT in predicting the risk of all-cause mortality in patients with DFUs.

## Materials and Methods

### Study Population

This retrospective cohort study enrolled 900 participants who were diagnosed with type 2 diabetes mellitus and DFUs according to the 2015 Diabetic Foot diagnostic criteria (12) in the First Affiliated Hospital of Wenzhou Medical University from 2015 to 2019. The exclusion criteria included lymphocytic leukemia, terminal malignancies, and hyperthyroidism, and those with missing data of ALB, total cholesterol TC, height, and weight. Finally, 771 patients were included in the study.

The study protocol was approved by the ethics committee of the First Affiliated Hospital of Wenzhou Medical University. The informed consent was exempted, due to the retrospective nature of the study.

### Data Collection and Grouping

The baseline data including demographic characteristics, anthropometric parameters, diabetes duration, hypertension, history of smoking, alcohol use and laboratory parameters including ALB, HbA1c, hemoglobin (Hb), creatinine, TC, triglyceride (TG), high-density lipoprotein cholesterol (HDL-C), and low-density lipoprotein cholesterol (LDL-C) were retrospectively extracted from individual medical records. For patients with multiple hospitalizations for DFUs, we only included the data of the first hospitalization. The endpoint for this study was all-cause mortality. Data regarding deaths were obtained in medical records or by telephone follow-up. Body mass index (BMI) was calculated as weight divided by height squared (kg/m^2^). Calculation of estimated glomerular filtration rate (eGFR), definition and grouping of smoking, alcohol use, and severe DFUs were as same as our previous study (13).

### Assessment of Nutritional Status

GNRI was calculated using formula as follows: GNRI = 1.489 × ALB (g/L) +41.7 × [weight (kg)/ideal body weight (kg)]. The ideal body weight was calculated as follows: for men: H −100–[(H−150)/4], for women: H −100–[(H−150)/2.5], where H indicates height (cm) (8). PNI was calculated using formula as follows: PNI = ALB (g/L) + 0.005 × lymphocyte count (/mm^3^) (9). CONUT was determined based on lymphocyte count, TC, and ALB as previously described (14).

### Statistical Analysis

The data are presented as mean ± standard deviation for normally distributed variables, while median and interquartile range for skewed variables, or *n* (%) for categorical variables. Differences were compared using student's *t*-test (normally distributed variables), Mann-Whitney U test (skewed variables) or Chi-squared test (categorical variables). Pearson (normally distributed variables), or spearman (skewed variables) correlation was used to assess the correlations between nutritional indices. The optimal cut-off values of nutritional indices for all-cause mortality were evaluated by the receiver operating characteristic (ROC) curves. The diagnostic performances of the optimal cut-off values were assessed using sensitivity, specificity, positive predictive value (PPV), negative predictive value (NPV), and accuracy. Kaplan-Meier survival curves and log-rank tests were used to compare the differences in overall survival (OS). The relationship between nutritional indices and all-cause mortality was analyzed by Cox proportional hazards regression. Variables with *P* < 0.1 in the unadjusted Cox regression analysis were included in the multivariable Cox regression analyses. BMI, ALB were excluded in the analyses of GNRI. Lymphocyte count and ALB were excluded in the analyses of PNI and CONUT, because they were used in the calculation of these nutritional indices. *P* values <0.05 were considered statistically significant for all tests. The statistical analyses were performed using SPSS (IBM, IL, USA) version 22. The pairwise comparison of ROC curves was performed using MedCalc version 20.019 (MedCalc Software Ltd, Ostend, Belgium).

## Results

### Analyses of Three Nutritional Indices

**Figure 1:**
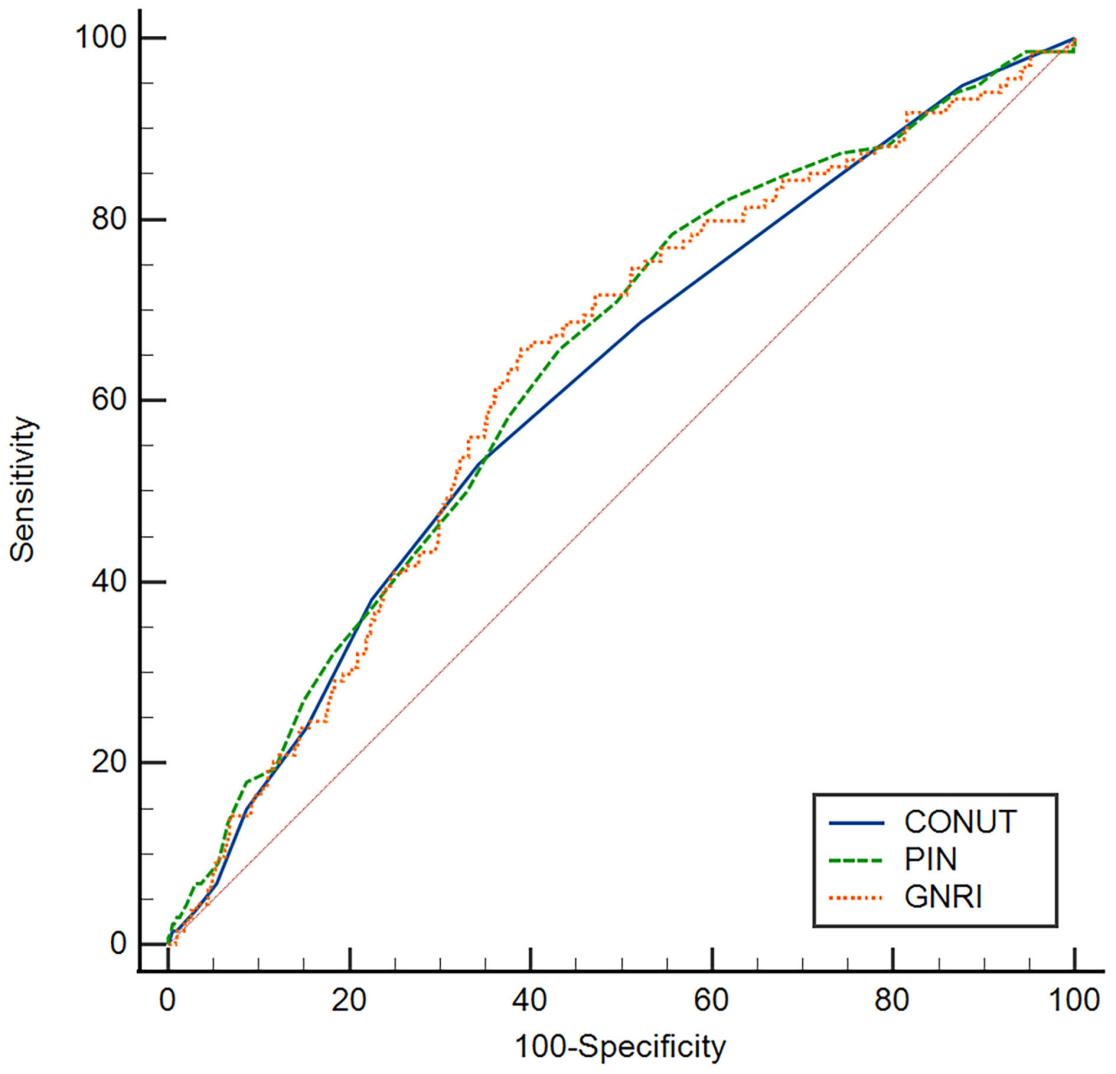
ROC curves of GNRI, PNI, and CONUT for predicting the all-cause mortality. The optimal cut-off values of GNRI, PNI, and CONUT were 93.1, 43.6, and 4.5, respectively. The AUC of GNRI was 0.630, 95% CI: 0.595–0.664, the AUC of PNI was 0.635, 95% CI: 0.600–0.669, the AUC of CONUT was 0.614, 95% CI: 0.578–0.648. ROC, receiver operating characteristic; AUC, area under ROC curve, GNRI, geriatric nutritional risk index; PNI, prognostic nutritional index; CONUT, controlling nutritional status.

The correlation coefficients were 0.75 between GNRI and PNI, −0.61 between GNRI and CONUT, −0.82 between PNI and CONUT, respectively (all *P* < 0.001). According to ROC analyses, patients with GNRI <93.1, PNI <43.6, and CONUT >4.5 were defined as high nutritional risk groups, others as low nutritional risk groups. There were 202 (26.2%) patients identified as at high risk of malnutrition by all three nutritional indices (Supplementary Figure 1) and 511 (66.3%) patients identified as at high risk of malnutrition by at least one of the nutritional indices. The area under ROC curve (AUC) of GNRI was 0.630, 95% CI: 0.595–0.664, the AUC of PNI was 0.635, 95% CI: 0.600–0.669, the AUC of CONUT was 0.614, 95% CI: 0.578–0.648 (Figure 1). The sensitivity, specificity, PPV, NPV, and accuracy of the cut-off values of nutritional indices are shown in Supplementary Table 1. Comparative analysis of ROC curves did not find significant differences among AUC values of three indices (all *p* > 0.05).

### Clinical Characteristics of the Study Population

Of the 771 patients included in the study, the BMI were 23.5 (21.6–25.9) (kg/m^2^), 84 (10.9%) were obese (BMI ≥28 kg/m^2^), 134 (17.4%) died during follow up. The clinical characteristics of patients classified as high nutritional risk groups and low nutritional risk groups based on the optimal cut-off values of GNRI, PNI, and CONUT are shown in Tables 1–3. Patients with high nutritional risk measured by any of the three nutritional indices had lower BMI, ALB, Lymphocyte, Hb, TC, TG, HDL-C, and LDL-C, longer diabetes duration, higher prevalence of severe DFUs, lower GNRI and PNI, higher CONUT than those with low nutritional risk. Furthermore, patients with high nutritional risk measured by GNRI had lower weight and higher HbA1c. Patients with high nutritional risk measured by PNI had longer diabetes duration and higher HbA1c, and were more likely to be men. Patients with high nutritional risk measured by CONUT had higher height, lower DBP, and were also more likely to be men (all *P* < 0.05).

**Table 1:** Baseline characteristics of participants with low and high nutritional risk according to GNRI.

**Characteristic**	**GNRI ≥93.1 (*N* = 435)**	**GNRI <93.1 (*N* = 336)**	***P*-value**
Male (%)	254 (58.4)	217 (64.6)	0.080
Age (years)	68 (60–75)	69 (59–76)	0.568
Height (cm)	163 (155–170)	164 (157–170)	0.330
Weight (kg)	66 (60–73)	58 (52–65)	<0.001
BMI (kg/m^2^)	24.8 (23.1–27.0)	21.9 (20.2–23.5)	<0.001
Smoking (%)	120 (27.6)	110 (32.7)	0.121
Alcohol use (%)	113 (26.0)	87 (25.9)	0.979
Diabetes duration (years)	10 (5–18)	10 (7–20)	0.008
Diabetic foot ulcer duration (days)	30 (15–60)	30 (12–60)	0.267
Severe DFUs (%)	183 (42.1)	236 (70.2)	<0.001
SBP (mmHg)	141 (128–157)	143 (126–161)	0.371
DBP (mmHg)	74 (67–82)	75 (66–85)	0.686
eGFR (EPI) (mL/min/1.73 m^2^)	84.3 (61.4–95.4)	79.5 (51.3–96.8)	0.182
ALB (g/L)	36.8 (34.4–39.2)	30.8 (27–33)	<0.001
Lymphocyte (× 10^9^/L)	1.6 (1.3–2.0)	1.4 (1.0–1.8)	<0.001
Hb (g/L)	120.7 ±17.1	106.8 ± 18.6	<0.001
HbA1c (%)	8.2 (7.2–9.8)	9.2 (7.8–11.4)	<0.001
TC (mmol/L)	4.19 (3.52–5.17)	3.71 (3.07–4.59)	<0.001
TG (mmol/L)	1.44 (1.03–1.98)	1.11 (0.82–1.60)	<0.001
HDL-C (mmol/L)	0.93 (0.79–1.13)	0.82 (0.66–1.05)	<0.001
LDL-C (mmol/L)	2.39 (1.81–3.10)	2.13 (1.59–2.79)	<0.001
GNRI	102.0 ± 6.5	85.6 ± 6.3	<0.001
PNI	45.3 ± 5.2	37.2 ± 5.6	<0.001
CONUT	3 (2–4)	5 (4–7)	<0.001

*BMI, body mass index; DFUs, diabetic foot ulcers; SBP, systolic blood pressure; DBP, diastolic blood pressure; eGFR, estimated glomerular filtration rate; ALB, albumin; Hb, hemoglobin; HbA1c, hemoglobin A1c; TC, total cholesterol; TG, triglyceride; HDL, high-density lipoprotein; LDL, low- density lipoprotein; GNRI, geriatric nutritional risk index; PNI, prognostic nutritional index; CONUT, controlling nutritional status*.

**Table 2:** Baseline characteristics of participants with low and high nutritional risk according to PNI.

**Characteristic**	**PNI ≥43.6 (*N* = 306)**	**PNI <43.6 (*N* = 465)**	***P*-value**
Male (%)	163 (53.3)	308 (66.2)	<0.001
Age (years)	67 (60–75)	69 (60–76)	0.326
Height (cm)	162 (155–170)	165 (157–170)	0.114
Weight (kg)	64 (56–70)	62 (56–69)	0.169
BMI (kg/m^2^)	24.1 (22.2–26.2)	23.1 (21.3–25.5)	<0.001
Smoking (%)	81 (26.5)	149 (32.0)	0.098
Alcohol use (%)	72 (23.5)	128 (27.5)	0.215
Diabetes duration (years)	10 (5–16)	10 (7–20)	<0.001
Diabetic foot ulcer duration (days)	30 (15–90)	30 (10–60)	0.020
Severe DFUs (%)	127 (41.5)	292 (62.8)	<0.001
SBP (mmHg)	143 (128–158)	142 (126–160)	0.682
DBP (mmHg)	76 (68–83)	74 (65–83)	0.073
eGFR (EPI) (mL/min/1.73 m^2^)	86.3 (66.2–97.0)	77.8 (51.1–94.8)	<0.001
ALB (g/L)	38.1 (36.2–40.1)	31.8 (28.2–34.0)	<0.001
Lymphocyte (× 10^9^/L)	1.9 (1.6–2.3)	1.3 (1.0–1.6)	<0.001
Hb (g/L)	124.2 ±16.1	108.3 ± 18.2	<0.001
HbA1c (%)	8.2 (7.3–9.7)	8.9 (7.5–11.1)	0.001
TC (mmol/L)	4.46 (3.59–5.47)	3.79 (3.10–4.55)	<0.001
TG (mmol/L)	1.47 (1.08–2.14)	1.15(0.85–1.65)	<0.001
HDL-C (mmol/L)	0.98 (0.83–1.15)	0.85 (0.67–1.06)	<0.001
LDL-C (mmol/L)	2.56 (1.87–3.30)	2.12 (1.65–2.78)	<0.001
GNRI	102.2 ± 7.9	89.9 ± 8.8	<0.001
PNI	48.1 ± 3.7	37.6 ± 4.6	<0.001
CONUT	2 (1–3)	5 (4–7)	<0.001

*BMI, body mass index; DFUs, diabetic foot ulcers; SBP, systolic blood pressure; DBP, diastolic blood pressure; eGFR, estimated glomerular filtration rate; ALB, albumin; Hb, hemoglobin; HbA1c, hemoglobin A1c; TC, total cholesterol; TG, triglyceride; HDL, high-density lipoprotein; LDL, low- density lipoprotein; GNRI, geriatric nutritional risk index; PNI, prognostic nutritional index; CONUT, controlling nutritional status*.

**Table 3:** Baseline characteristics of participants with low and high nutritional risk according to CONUT.

**Characteristic**	**CONUT ≤4.5 (*N* = 482)**	**CONUT >4.5 (*N* = 289)**	***P*-value**
Male (%)	266 (55.2)	205 (70.9)	<0.001
Age (years)	68 (61–76)	68 (59–76)	0.669
Height (cm)	162 (155–170)	165 (158–170)	0.004
Weight (kg)	63 (56–70)	62 (55–70)	0.612
BMI (kg/m^2^)	23.9 (21.9–26.0)	23.0 (20.9–25.4)	0.002
Smoking (%)	139 (28.8)	91 (31.5)	0.436
Alcohol use (%)	123 (25.5)	77 (26.6)	0.730
Diabetes duration (years)	10 (5–17)	10 (7–20)	0.011
Diabetic foot ulcer duration (days)	30 (13–90)	30 (14–60)	0.528
Severe DFUs (%)	220 (45.6)	199 (68.9)	<0.001
SBP (mmHg)	143 (128–160)	141 (124–158)	0.132
DBP (mmHg)	76 (68–84)	73 (64–82)	0.003
eGFR (EPI) (mL/min/1.73 m^2^)	84.0 (61.4–95.8)	79.5 (51.8–95.8)	0.140
ALB (g/L)	36.2 (33.5–38.7)	29.7 (26.6–33.2)	<0.001
Lymphocyte (× 10^9^/L)	1.7 (1.4–2.1)	1.1 (0.8–1.5)	<0.001
Hb (g/L)	120.2 ± 17.1	105.4 ± 18.6	<0.001
HbA1c (%)	8.6 (7.5–10.3)	8.7 (7.4–11.1)	0.229
TC (mmol/L)	4.51 (3.76–5.40)	3.33 (2.75–3.97)	<0.001
TG (mmol/L)	1.44 (1.06–2.01)	1.05 (0.80–1.51)	<0.001
HDL-C (mmol/L)	0.98 (0.79–1.15)	0.80 (0.61–0.98)	<0.001
LDL-C (mmol/L)	2.64 (1.99–3.27)	1.84 (1.45–2.38)	<0.001
GNRI	98.9 ± 8.5	88.0 ± 9.6	<0.001
PNI	45.2 ± 4.9	35.9 ± 5.2	<0.001
CONUT	3 (2–4)	6 (5–8)	<0.001

*BMI, body mass index; DFUs, diabetic foot ulcers; SBP, systolic blood pressure; DBP, diastolic blood pressure; eGFR, estimated glomerular filtration rate; ALB, albumin; Hb, hemoglobin; HbA1c, hemoglobin A1c; TC, total cholesterol; TG, triglyceride; HDL, high-density lipoprotein; LDL, low- density lipoprotein; GNRI, geriatric nutritional risk index; PNI, prognostic nutritional index; CONUT, controlling nutritional status*.

Patients with severe DFUs (Wagner grade score ≥3) had a higher prevalence of high nutritional risk than those with no severe DFUs (Supplementary Table 2) (all *P* < 0.001).

### Kaplan-Meier Curves for OS

**Figure 2:**
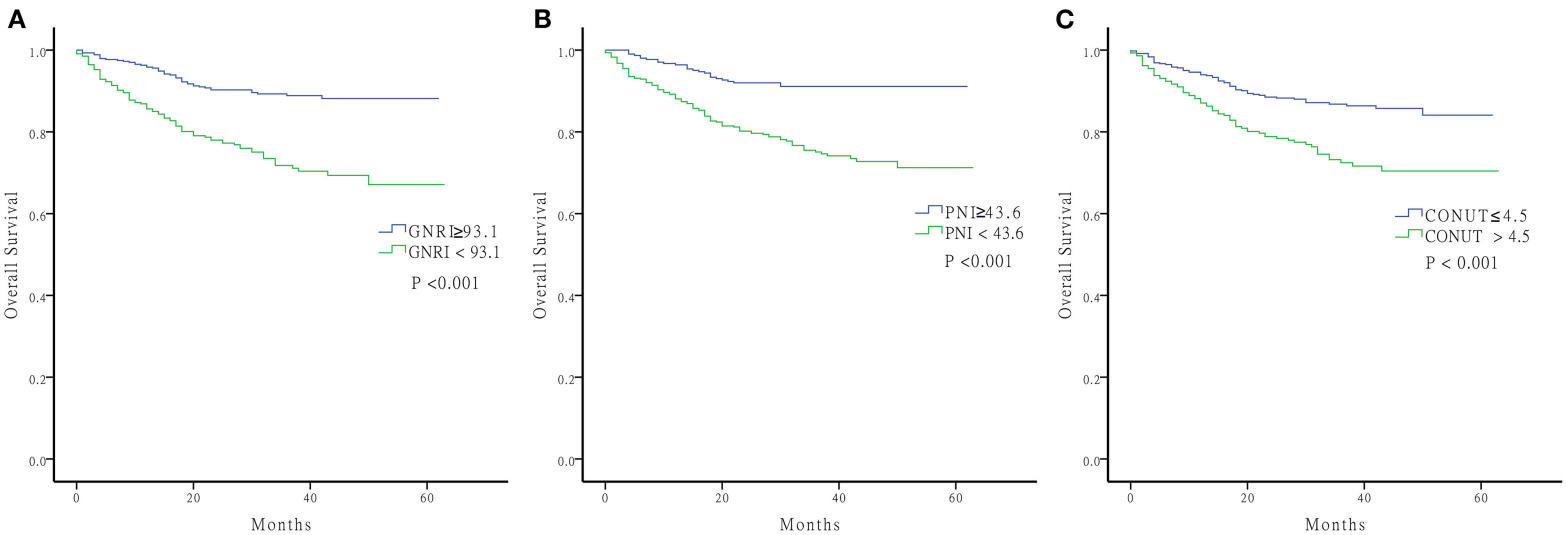
Kaplan-Meier curve of OS. **(A)** GNRI ≥93.1 and GNRI <93.1 **(B)** PNI ≥43.6 and PNI <43.6 **(C)** CONUT ≤ 4.5 and CONUT >4.5 OS, Overall Survival; GNRI, geriatric nutritional risk index; PNI, prognostic nutritional index; CONUT, controlling nutritional status.

Log-rank tests of the Kaplan Meier curves indicated that patients with high nutritional risk measured by the three nutritional indices had lower OS rates compared to those with low nutritional risk (Figure 2) (all *P* < 0.001). The overall cumulative survival rates at 1, 3, and 5 years are shown in Supplementary Table 3.

### Unadjusted and Multivariate Cox Regression Analyses for All-Cause Mortality

The unadjusted and multivariable-adjusted Cox regression analyses were performed to evaluate the relationship between three nutritional indices and all-cause mortality (Table 4). In addition to GNRI, PNI, and CONUT, the unadjusted Cox regression analyses also found that age, weight, BMI, SBP, severe DFUs, ALB, lymphocyte, Hb, and eGFR were significantly associated with all-cause mortality (Supplementary Table 4). The multivariable Cox regression revealed that low GNRI (adjusted HR 2.01, 95% CI: 1.37–2.96, *P* < 0.001), low PNI (adjusted HR 2.04, 95% CI: 1.29–3.23, *P* = 0.002), and high CONUT (adjusted HRs 1.54, 95% CI: 1.07–2.23, *P* = 0.021) were independently associated with high all-cause mortality. In subgroup analyses, according to the severity of DFUs, the observed associations among three nutritional indices and all-cause mortality remained significant in patients with no severe DFUs. However, in patients with severe DFUs, only the association between low GNRI and high all-cause mortality remained significant after adjusting for confounding factors.

**Table 4:** Unadjusted and multivariate Cox regression analyses for all-cause mortality.

	**Unadjusted HR (95% CI)**	***P*-value**	**Adjusted HR (95% CI)**	***P*-value**
**Total**				
**GNRI**				
Low nutritional risk (≥93.1)	Ref	_	Ref	_
nutritional risk (<93.1)	2.85 (2.00–4.08)	<0.001	2.01 (1.37–2.96) ^a^	<0.001
**PNI**				
Low nutritional risk (≥43.6)	Ref	_	Ref	_
High nutritional risk (<43.6)	3.11 (2.02–4.77)	<0.001	2.04 (1.29–3.23) ^b^	0.002
**CONUT**				
Low nutritional risk (≤4.5)	Ref	_	Ref	_
High nutritional risk (>4.5)	2.12 (1.51–2.98)	<0.001	1.54 (1.07–2.23)^b^	0.021
**Severe DFUs**				
**GNRI**				
Low nutritional risk (≥93.1)	Ref	_	Ref	_
nutritional risk (<93.1)	1.96 (1.24–3.07)	0.004	2.07 (1.28–3.35)^c^	0.003
**PNI**				
Low nutritional risk (≥43.6)	Ref	_	Ref	_
High nutritional risk (<43.6)	1.92 (1.14–3.22)	0.014	1.51 (0.87–2.60)^d^	0.141
**CONUT**				
Low nutritional risk (≤4.5)	Ref	_	Ref	_
High nutritional risk (>4.5)	1.43 (0.94–2.17)	0.095	1.28 (0.82–2.00)^d^	0.285
**No severe DFUs**				
**GNRI**				
Low nutritional risk (≥93.1)	Ref	_	Ref	_
nutritional risk (<93.1)	3.94 (2.18–7.11)	<0.001	2.19 (1.14–4.22)^c^	0.019
**PNI**				
Low nutritional risk (≥43.6)	Ref	_	Ref	_
High nutritional risk (<43.6)	5.37 (2.50–11.53)	<0.001	3.71 (1.59–8.63)^d^	0.002
**CONUT**				
Low nutritional risk (≤4.5)	Ref	_	Ref	_
High nutritional risk (>4.5)	3.36 (1.87–6.03)	<0.001	2.39 (1.28–4.48)^d^	0.006

a *The multivariable Cox regression was adjusted for risk factors including age, SBP, severe DFUs, lymphocyte, Hb, eGFR*.

b *The multivariable Cox regression was adjusted for risk factors including age, SBP, severe DFUs, BMI, Hb, eGFR*.

c *The multivariable Cox regression was adjusted for risk factors including age, SBP, lymphocyte, Hb, eGFR*.

d *The multivariable Cox regression was adjusted for risk factors including age, SBP, BMI, Hb, eGFR. GNRI, geriatric nutritional risk index; PNI, prognostic nutritional index; CONUT, controlling nutritional status. Ref, reference; DFUs, diabetic foot ulcers; SBP, systolic blood pressure; eGFR, estimated glomerular filtration rate; Hb, hemoglobin*.

## Discussion

To the best of our knowledge, the present study is the first study to investigate the predictive value of three nutritional indices concurrently in patients with DFUs. The present study suggested that patients with high nutritional risk defined by any of three objective nutritional indices, GNRI, PNI, and CONUT, had lower OS rates. Low GNRI, low PNI, and high CONUT were independently associated with high all-cause mortality, even after adjusting for confounding variables by multivariate Cox regression analysis. No significant difference was found among the predictive capability of GNRI, PNI, and CONUT by comparative analysis of ROC curves in total population with DFUs. In subgroup analyses, low GNRI, but not low PNI or high CONUT, predicted higher all-cause mortality in patients with severe DFUs, while all of the three indices persisted as independent prognostic factors in patients with no severe DFUs.

There is no generally accepted set of criteria for malnutrition, and the prevalence of malnutrition varies depending on the nutritional screening methods. Although significant correlations were found among GNRI, PNI, and CONUT, different parameters in each index might influence the prevalence of malnutrition. A total of 511 (66.3%) patients were identified as with high nutritional risk by at least one of the nutritional indices, indicating that malnutrition was a common occurrence in patients with DFUs. There were many potential contributing factors to the risk of malnutrition among patients with DFUs. The main reasons for developing malnutrition included decreased nutritional intake, increased energy and protein requirements, increased losses and inflammation (15). Response to trauma or infection related to DFUs might alter metabolism, appetite, and absorption, leading to insufficient intake. Drug-related side effects, such as antibiotics and painkillers, may also cause anorexia (15). Moreover, reduced mobility in patients with DFUs may cause severe catabolism then reduce muscle protein synthesis (16). Furthermore, patients with DFUs were accompanied by a sustained inflammatory state (17), which could contribute to hypoalbuminemia by increasing capillary permeability and promoting protein degradation (18).

Previous studies reported that nutritional screening tools, such as subjective global assessment, mini nutritional assessment, Haute Autorité de Santé criteria, and GNRI could identify DFUs at risk of malnutrition (5, 11, 19, 20). However, only one study with a small sample size demonstrated that malnutrition determined by GNRI was associated with all-cause mortality in patients undergoing amputations due to DFUs (11). The present study demonstrated the prognostic value of three objective nutritional indices, GNRI, PNI, and CONUT, in a relatively large cohort of patients with DFUs.

Nutritional status has been reported to be an important predicted indicator of mortality in various diseases (7, 21–23). Long-term chronic disease, including diabetes, results in malnutrition, which may exacerbate the disease and contribute to an unfavorable prognosis (24). The parameters in each index might also explain the associations of nutritional indices with all-cause mortality. Albumin was the common component of three nutritional indices. Albumin reflected nutritional status and systemic inflammation (18). Hypoalbuminemia was associated with mortality in patients regardless of the implicated disease, even in a healthy population (25). Lymphocytes reflected the immune regulatory response (26). Lymphocyte count and leukocyte ratios that mainly included lymphocyte were predictors of mortality in patients with type 2 diabetes and DFUs (26, 27). Lower BMI was found to be associated with mortality in patients with diabetes and DFUs in recent studies (28, 29), which was consistent with our finding: BMI was negatively associated with all-cause mortality in the unadjusted Cox regression analyses. Patients with type 2 diabetes are usually overweight or obese. However, the median BMI of patients in the study was 23.5 (kg/m^2^), which was relativity lower than the mean BMI (around 25 kg/m^2^) of patients with type 2 diabetes in China (30). 10.9% of patients in this study were obese (BMI ≥28 kg/m^2^), which were lower than that (16.4% for obesity) of general populations in China (31). The above findings suggested that the prevalence of malnutrition was higher among patients with type 2 diabetes and DFUs than those with type 2 diabetes but without DFUs, and the general population.

Since most malnutrition can be caused due to diseases and the risk of malnutrition increases with the severity of disease (15), subgroup analyses were performed according to the severity of DFUs. In subgroup analyses, nutritional status affected all-cause mortality more strongly in patients with no severe DFUs than those with severe DFUs. This discrepancy might be due to the differences in patients' characteristics. In this study, patients with severe DFUs had a higher prevalence of high nutritional risk measured by any of the three nutritional indices, and vice versa. However, the underlying mechanism by which mortality of severe DFUs was less affected by nutritional status, needs to be clarified by further studies. GNRI was the only independent prognostic factor in patients with severe DFUs. GNRI included ALB, height, and weight. PNI included ALB and lymphocyte count. CONUT was similar to the PNI, except for an additional parameter: TC. However, TC was not associated with mortality in unadjusted Cox regression analyses. GNRI contained both anthropometric factors and serum factors, while CONUT and PNI contained only serum factors. Therefore, GNRI was considered to be a better nutritional screening tool than PNI and CONUT because it was multidimensional (7).

This study has two strengths. First, the sample size of this study is relatively large. Second, the nutritional indices used in this study were objective and simple. There were many screening tools for malnutrition indices, such as subjective global assessment, which is a multidimensional screening tool. It subjectively classified patients based on medical history and physical examination, and has no numerical scoring system (5, 32). Subjective global assessment is not objective, therefore, it is not suitable for intervention and follow-up studies (32). Subjective global assessment requires detailed training of medical staff and cooperation of patients, whereas the three indices in this study can be conveniently used in a clinical setting. This study also has several limitations. First, this is a single center study. Therefore, the results may not be applicable to general patients with DFUs. Second, we only evaluated the nutritional indices at baseline, without dynamic observation of indices during follow-up. Third, our analysis is limited to all-cause mortality, not disease-specific mortality. Fourth, another limitation is the lack of sufficient information on concomitant cardiovascular diseases. The present findings need to be confirmed by further well-designed studies in different settings and cohorts with a dynamic observation of nutritional indices.

In conclusion, the present study demonstrated that malnutrition was common in patients with DFUs. Three objective and simple nutritional indices, namely GNRI, PNI, and CONUT were powerful predictors of mortality in patients with DFUs. Routine screening for malnutrition using any of the three nutritional indices might be a simple and effective way to identify high-risk patients with DFUs. GNRI can be used as an independent prognostic indicator in patients with severe DFUs. Early nutritional interventions might help to improve the prognosis of patients with high nutritional risk, which needs to be clarified by further studies.

## Data Availability Statement

The raw data supporting the conclusions of this article will be made available by the authors, without undue reservation.

## Ethics Statement

The studies involving human participants were reviewed and approved by the Ethics Committee of the First Affiliated Hospital of Wenzhou Medical University. Written informed consent for participation was not required for this study in accordance with the national legislation and the institutional requirements.

## Author Contributions

JH, HZ, and Q-QH: study concept and design. F-FJ and Z-RX: acquisition of data. JH and W-YL: analysis and interpretation of data. JH: drafting of the manuscript. W-YL, Q-QH, XH, F-XS, and HZ: critical revision of the manuscript for important intellectual content. All authors read and approved the final manuscript.

## Funding

This work was supported by the National Natural Science Foundation of China (81900737), the Basic Scientific Research Program of Wenzhou Medical University, China (KYYW202015).

## Conflict of Interest

The authors declare that the research was conducted in the absence of any commercial or financial relationships that could be construed as a potential conflict of interest.

## Publisher's Note

All claims expressed in this article are solely those of the authors and do not necessarily represent those of their affiliated organizations, or those of the publisher, the editors and the reviewers. Any product that may be evaluated in this article, or claim that may be made by its manufacturer, is not guaranteed or endorsed by the publisher.

## Supplementary Material

Supplementary Figure 1 Patients [*n* (%)] defined as high nutritional risk (GNRI <93.1, PNI <43.6 or CONUT >4.5) were shown inside the circle, and patients [*n* (%)] defined as low nutritional risk were shown outside the circle. GNRI, geriatric nutritional risk index; PNI, prognostic nutritional index; CONUT, controlling nutritional status.

Supplementary Table 1 Diagnostic performances of optimal cut-off values of nutritional indices.

Supplementary Table 2 Prevalence of high nutritional risk according to the severity of DFUs.

Supplementary Table 3 Cumulative overall survival of participants categorized by GNRI, PNI, and CONUT.

Supplementary Table 4 Unadjusted Cox regression analyses for all-cause mortality.

## Abbreviations

ALB: albumin
AUC: area under ROC curve
BMI: body mass index
CONUT: controlling nutritional status
DBP: diastolic blood pressure
DFUs: diabetic foot ulcers
eGFR: estimated glomerular filtration rate
GNRI: geriatric nutritional risk index
HbA1c: hemoglobin A1c
Hb: hemoglobin
HDL: high-density lipoprotein
LDL: low-density lipoprotein
NPV: negative predictive value
OS: overall survival
PNI: prognostic nutritional index
PPV: positive predictive value
Ref: reference
ROC: receiver operating characteristic
SBP: systolic blood pressure
TC: total cholesterol
TG: triglyceride.
