# Supplementary figures and images for: Three Nutritional Indices Are Effective Predictors of Mortality in Patients With Type 2 Diabetes and Foot Ulcers
Source: Front Nutr. 2022 Mar 15;9:851274. doi: 10.3389/fnut.2022.851274 (PMC8965352; doi:10.3389/fnut.2022.851274)

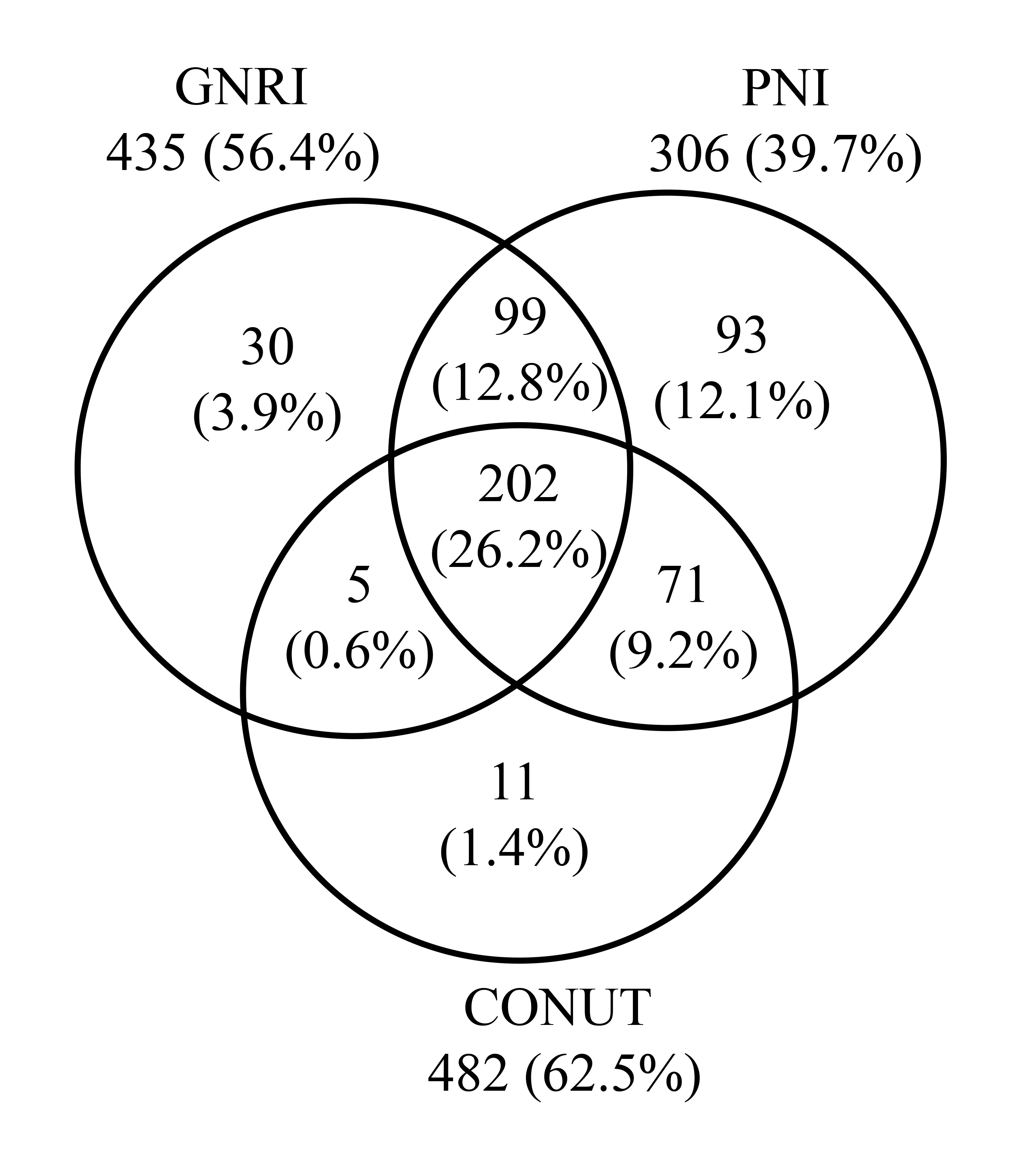

Supplement: Supplementary Figure 1 — Patients [n (%)] defined as high nutritional risk (GNRI <93.1, PNI <43.6 or CONUT >4.5) were shown inside the circle, and patients [n (%)] defined as low nutritional risk were shown outside the circle. GNRI, geriatric nutritional risk index; PNI, prognostic nutritional index; CONUT, controlling nutritional status. [file Image_1.TIF]
