# Supplementary material for: Three Nutritional Indices Are Effective Predictors of Mortality in Patients With Type 2 Diabetes and Foot Ulcers
Source: Front Nutr. 2022 Mar 15;9:851274. doi: 10.3389/fnut.2022.851274 (PMC8965352; doi:10.3389/fnut.2022.851274)
Supplement: Supplementary Table 1 — Diagnostic performances of optimal cut-off values of nutritional indices. [file Table_1.docx]

Supplemental Table 1 Diagnostic performances of optimal cut-off values of nutritional indices

| Cut-off values | Sensitivity (%) | Specificity (%) | PPV (%) | NPV (%) | Accuracy (%) |
| --- | --- | --- | --- | --- | --- |
| GNRI: 93.1 | 65.7 | 61.1 | 26.2 | 82.6 | 61.9 |
| PNI: 43.6 | 80.6 | 44.0 | 23.2 | 91.5 | 50.3 |
| CONUT: 4.5 | 53.0 | 65.8 | 24.6 | 86.9 | 63.6 |

GNRI: geriatric nutritional risk index; PNI: prognostic nutritional index; CONUT: controlling nutritional status; PPV: positive predictive value; NPV: negative predictive value.
