# Supplementary material for: Three Nutritional Indices Are Effective Predictors of Mortality in Patients With Type 2 Diabetes and Foot Ulcers
Source: Front Nutr. 2022 Mar 15;9:851274. doi: 10.3389/fnut.2022.851274 (PMC8965352; doi:10.3389/fnut.2022.851274)
Supplement: Supplementary Table 2 — Prevalence of high nutritional risk according to the severity of DFUs. [file Table_2.docx]

Supplemental Table 2 Prevalence of high nutritional risk according to the severity of DFUs

| Characteristic | No severe DFUs  (N = 352) | severe DFUs  (N = 419) | P-value |
| --- | --- | --- | --- |
| GNRI < 93.1 (%) | 100 (28.4) | 236 (56.3) | < 0.001 |
| PNI < 43.6 (%) | 173 (49.1) | 292 (69.7) | < 0.001 |
| CONUT > 4.5 (%) | 90 (25.6) | 199 (47.5) | < 0.001 |

DFUs: diabetic foot ulcers; GNRI: geriatric nutritional risk index; PNI: prognostic nutritional index; CONUT: controlling nutritional status.
