# Supplementary material for: Three Nutritional Indices Are Effective Predictors of Mortality in Patients With Type 2 Diabetes and Foot Ulcers
Source: Front Nutr. 2022 Mar 15;9:851274. doi: 10.3389/fnut.2022.851274 (PMC8965352; doi:10.3389/fnut.2022.851274)
Supplement: Supplementary Table 3 — Cumulative overall survival of participants categorized by GNRI, PNI, and CONUT. [file Table_3.docx]

Supplemental Table 3 Cumulative overall survival of participants categorized by GNRI, PNI, and CONUT

|  | 1 Years OS | 3 Years OS | 5 Years OS |
| --- | --- | --- | --- |
| GNRI |  |  |  |
| Low nutritional risk (≥93.1) | 95.8% | 88.9% | 88.2% |
| High nutritional risk (<93.1) | 85.6% | 71.8% | 67.1% |
| PNI |  |  |  |
| Low nutritional risk (≥43.6) | 96.4% | 91.1% | 91.1% |
| High nutritional risk (<43.6) | 88.1% | 75.1% | 71.2% |
| CONUT |  |  |  |
| Low nutritional risk (≤ 4.5) | 94.0% | 86.8% | 84.1% |
| High nutritional risk (> 4.5) | 87.0% | 72.5% | 70.4% |

OS: overall survival.
