# Supplementary material for: Three Nutritional Indices Are Effective Predictors of Mortality in Patients With Type 2 Diabetes and Foot Ulcers
Source: Front Nutr. 2022 Mar 15;9:851274. doi: 10.3389/fnut.2022.851274 (PMC8965352; doi:10.3389/fnut.2022.851274)
Supplement: Supplementary Table 4 — Unadjusted Cox regression analyses for all-cause mortality. [file Table_4.docx]

Supplemental Table 4 Unadjusted Cox regression analyses for all-cause mortality

| Characteristic | Unadjusted HR (95%CI) | P-value |
| --- | --- | --- |
| Male (vs. Female) | 1.19 (0.83-1.70) | 0.337 |
| Age (per year increase) | 1.07 (1.05-1.09) | < 0.001 |
| Height (per 1-cm increase) | 0.99 (0.97-1.01) | 0.143 |
| Weight (per 1-kg increase) | 0.98 (0.96-1.00) | 0.009 |
| BMI (per 1-kg/m^2^ increase) | 0.94 (0.89-0.99) | 0.021 |
| Smoking (vs. non-smoking) | 0.96 (0.66-1.39) | 0.823 |
| Alcohol use (vs. no alcohol consumption) | 0.95 (0.64-1.40) | 0.779 |
| Diabetes duration (per year increase) | 1.01 (0.99-1.02) | 0.383 |
| Diabetic foot duration (per day increase) | 1.00 (1.00-1.00) | 0.234 |
| Severe DFUs (vs. no severe DFUs) | 1.92 (1.34-2.75) | < 0.001 |
| SBP (per 1-mmHg increase) | 1.01 (1.00-1.02) | 0.014 |
| DBP (per 1-mmHg increase) | 0.99 (0.98-1.00) | 0.180 |
| eGFR (EPI) (per 1-mL/min/1.73m2 increase) | 0.98 (0.97-0.99) | < 0.001 |
| ALB (per 1-g/L increase) | 0.94 (0.92-0.97) | < 0.001 |
| Lymphocyte (per 1×10^9^/L increase) | 0.56 (0.42-0.77) | < 0.001 |
| Hb (per 1-g/L increase) | 0.97 (0.96-0.98) | < 0.001 |
| HbA1c (per 1% increase) | 0.95 (0.87-1.04) | 0.284 |
| TC (per 1-mmol/L increase) | 0.98 (0.87-1.10) | 0.746 |
| TG (per 1-mmol/L increase) | 1.02 (0.88-1.17) | 0.832 |
| HDL-C (per1-mmol/L increase) | 0.64 (0.36-1.14) | 0.128 |
| LDL-C (per1-mmol/L increase) | 1.02 (0.86-1.21) | 0.826 |

Abbreviations as in Table 1
